# Optic neuritis following diphtheria, tetanus, pertussis, and inactivated poliovirus combined vaccination: a case report

**DOI:** 10.1186/s13256-018-1903-9

**Published:** 2018-11-30

**Authors:** Preston O’Brien, Robert W. Wong

**Affiliations:** 1 Austin Retina Associates, 801 W. 38th St, Suite 200, Austin, TX 78705 USA; 2 Department of Surgery and Perioperative Services, Dell Medical School, Austin, TX USA

**Keywords:** Diphtheria, Tetanus, Pertussis, Virus, Optic neuritis

## Abstract

**Background:**

Diphtheria, tetanus, pertussis, and inactivated poliovirus combined vaccine is widely used in young children as part of a series of immunizations before they start attending school. Case studies of demyelinating conditions following administration of diphtheria, tetanus, pertussis, and polio vaccine have been reported, but none so far resulting in optic neuritis. This report further contributes to the database of central nervous system demyelinating conditions affiliated with receipt of vaccines.

**Case presentation:**

A previously healthy 27-year-old Hispanic man presented to an emergency department with headache, periorbital pressure, pain with ocular movements, and intermittent blurred vision that developed 1 day after administration of the diphtheria, tetanus, pertussis, and inactivated poliovirus combined vaccine. A diagnosis of optic neuritis was made via ophthalmic examination with fundus photography and automated Humphrey visual field analysis. His vision recovered following treatment with high-dose intravenously administered methylprednisolone followed by a tapered dose of orally administered prednisolone.

**Conclusions:**

Although the association between immunizations and the onset of central nervous system demyelinating conditions is well documented, this report, to the best of our knowledge, is the first case of optic neuritis following diphtheria, tetanus, pertussis, and inactivated poliovirus combined vaccination. Inclusion of this case report in the medical community will allow for broader understanding of possible conditions that may present shortly after receipt of vaccination.

## Background

Diphtheria, tetanus, pertussis, and inactivated poliovirus combined vaccine (DTaP-IPV) is widely used in young children as part of a series of immunizations before they start attending school. Although clinical trials have shown an excellent safety profile [1], there have been reports of encephalitis, angioneurotic edema, seizures, and serious local reactions following its administration [1, 2]. Although cases of central nervous system (CNS) demyelinating conditions following DTaP-IPV vaccine have been reported [3], to the best of our knowledge, we present the first case of optic neuritis.

## Case presentation

A 27-year-old Hispanic man with no significant past medical history presented to an emergency department with a 5-day history of headache, pain with ocular movements, and intermittent blurred vision starting 1 day after being immunized with DTaP-IPV. Magnetic resonance imaging and a magnetic resonance venogram of his brain were unremarkable. A lumbar puncture revealed a normal opening pressure and cerebrospinal fluid studies were positive for myelin basic protein but negative for oligoclonal bands and neuromyelitis optica autoantibody serology.

**Fig. 1:**
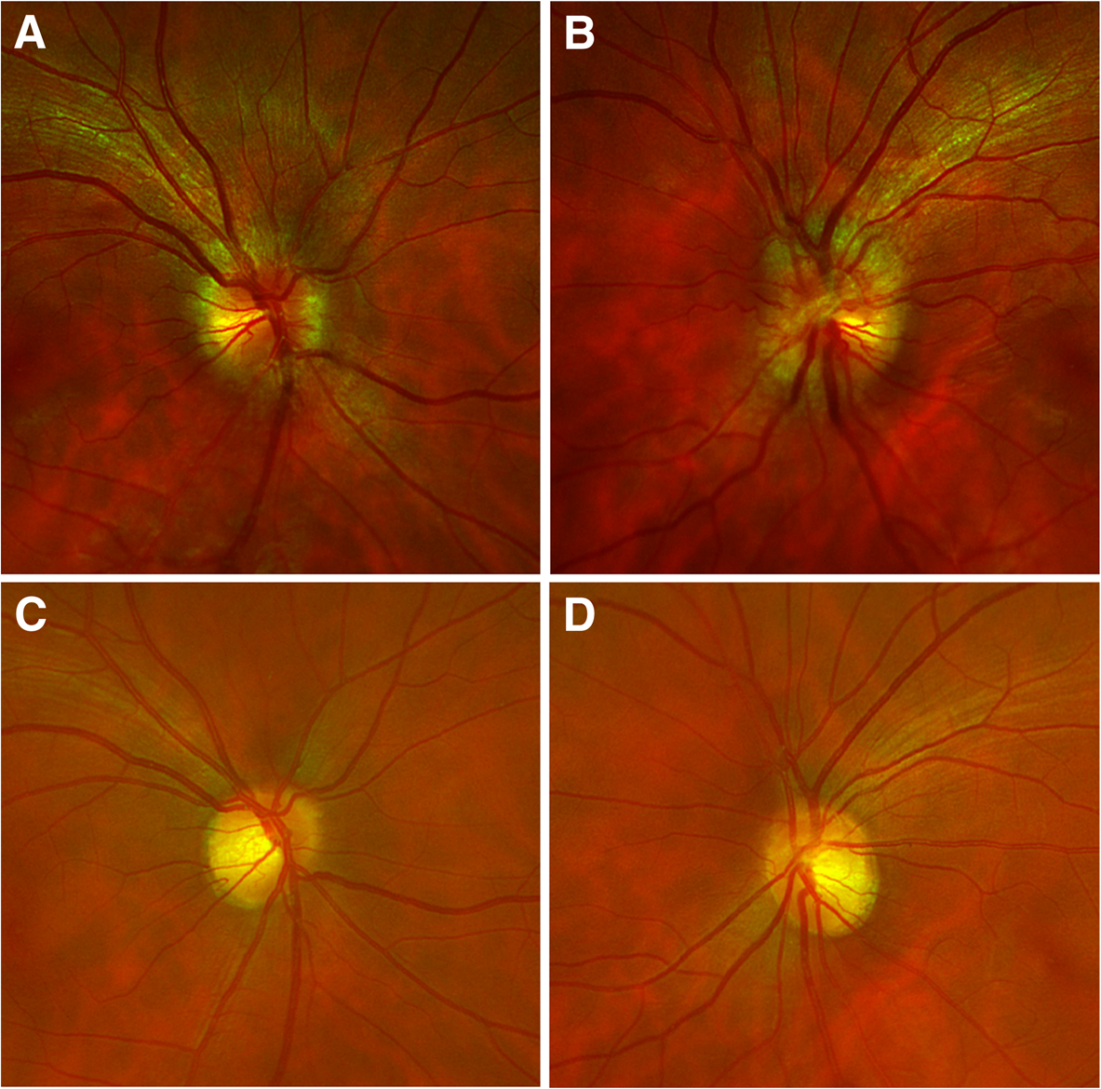
Color fundus photography of the optic nerve 5 days after initial presentation when vision dropped to counting fingers at 30.5 cm (1 foot) in both eyes of (**a**) right eye and (**b**) left eye. Resolution of optic nerve hyperemia seen on the right eye (**c**) and the left eye (**d**) after treatment with corticosteroids

On examination, his best corrected vision was 20/100 in his right eye and 20/70 in his left eye. Intraocular pressures, pupil examination, ocular alignment, and extraocular movements were normal. Confrontational visual fields were restricted in both eyes. Posterior segment examination showed optic nerve swelling and hyperemia in both eyes (Fig. 1) and two microaneurysms in the mid periphery of his left eye. No evidence of vitritis, retinal vasculitis, or choroiditis was seen in either eye.

**Fig. 2:**
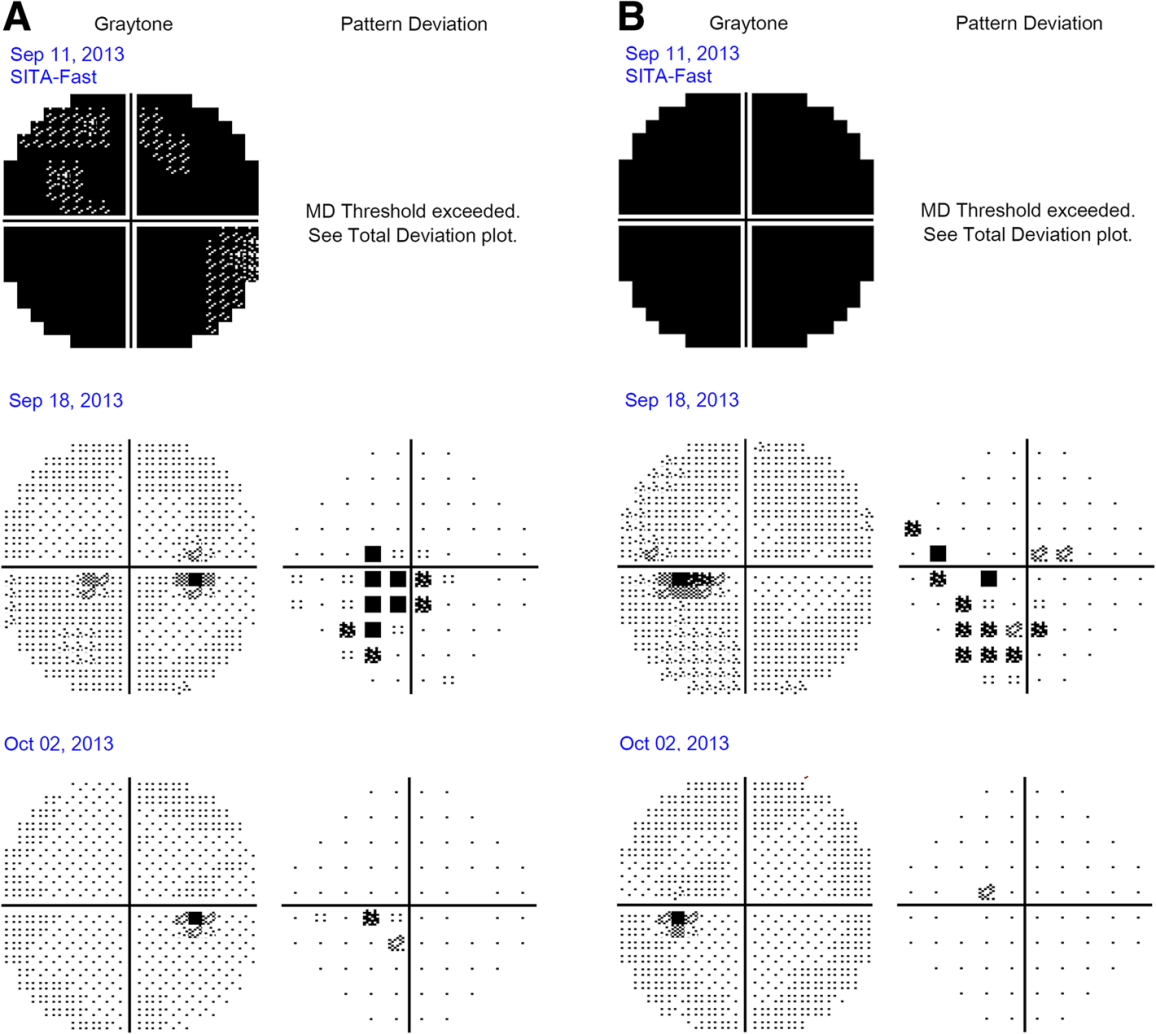
Automated 30–2 Humphrey visual field testing using Swedish Interactive Testing Algorithm-Fast protocol of (**a**) the right eye and (**b**) the left eye showing gray tone and pattern deviation. The patient was started on intravenously administered Solu-Medrol (methylprednisolone) on September 11, 2013 (*top line*). Follow-up perimetry 1 week (*middle line*) and 3 weeks (*bottom line*) after the initiation of systemic corticosteroids showing improvement and resolution of the visual field deficits. *MD* mean deviation, *SITA* Swedish Interactive Testing Algorithm

Serum laboratory testing showed elevated glycated hemoglobin (A1C) at 6.9%, aspartate aminotransferase (AST), and alanine aminotransferase (ALT). Other liver tests including bilirubin, alkaline phosphatase, and hepatitis serologies were normal. Tests for infectious and inflammatory etiologies including angiotensin-converting enzyme (ACE), lysozyme, antinuclear antibody (ANA), cytoplasmic antineutrophil cytoplasmic antibodies (c-ANCA), perinuclear antineutrophil cytoplasmic antibodies (p-ANCA), lupus panel, rapid plasma reagin (RPR), fluorescent treponemal antibody absorption (FTA-ABS), chest X-ray, and QuantiFERON Gold assay, which were normal. Over the next 5 days, his vision declined to counting fingers at 30.5 cm (1 foot) in both eyes. A relative afferent pupil defect and dyschromatopsia developed on the left. Automated Humphrey visual field (HVF) testing demonstrated global depression in both eyes (Fig. 2).

He was diagnosed as having DTaP-IPV vaccination-related optic neuritis and started on intravenously administered Solu-Medrol (methylprednisolone). One week later, his headache resolved and vision improved to 20/20 in his right eye and 20/25 in his left eye with less optic nerve hyperemia and swelling. He was discharged on a prednisone taper and an orally administered diabetic medication. One month later, his vision improved to 20/20 with resolution of the optic neuritis without residual visual field deficit in both eyes.

## Discussion

In 2008, the DTaP-IPV vaccine was licensed and indicated for use in children of 4–6 years in age. From 2009 to 2012, a large-scale trial monitoring for adverse events found no significant increased risk of meningitis or encephalitis following DTaP-IPV [1]. Although the overall risk of developing a demyelinating CNS syndrome after vaccination is relatively low (estimated to be 0.1%), it is not negligible [3]. Molecular mimicry from the viral proteins or the adjuvants used in the preparation of the vaccine have been suspected in the development of demyelinating disease following vaccination [3, 4]. Molecular mimicry occurs when similarities exist between proteins of viruses used in vaccinations and the components of CNS myelin which may disrupt self-tolerance and cause production of autoantibodies resulting in CNS inflammation including optic neuritis [3, 5]. Our case is consistent with other cases of post-vaccination optic neuritis, most of which develop 1–3 weeks after vaccination, typical of an immune-triggered mechanism [3].

In most cases, symptoms of optic neuritis were mostly resolved after treatment with steroids such as intravenously administered methylprednisolone followed by tapered oral prednisolone for several weeks [3, 5]. Early recognition of ocular signs and symptoms of optic neuritis following DTaP-IPV vaccination may lead to prompt treatment and preserved vision.

## Conclusions

Although the association between immunizations and the onset of CNS demyelinating conditions is well documented, this report, to the best of our knowledge, is the first case of optic neuritis following DTaP-IPV vaccination. Inclusion of this case report in the medical community will allow for broader understanding of possible conditions that may present shortly after receipt of vaccination.

## Abbreviations

A1C: Glycated hemoglobin
ACE: Angiotensin-converting enzyme
ALT: Alanine aminotransferase
ANA: Antinuclear antibody
AST: Aspartate aminotransferase
c-ANCA: Cytoplasmic antineutrophil cytoplasmic antibodies
CNS: Central nervous system
DTaP-IPV: Diphtheria, tetanus, pertussis and inactivated poliovirus combined vaccine
FTA-ABS: Fluorescent treponemal antibody absorption
p-ANCA: Perinuclear antineutrophil cytoplasmic antibodies
RPR: Rapid plasma reagin
